# DNA Damage-Induced Neurodegeneration in Accelerated Ageing and Alzheimer’s Disease

**DOI:** 10.3390/ijms22136748

**Published:** 2021-06-23

**Authors:** Heling Wang, Sofie Lautrup, Domenica Caponio, Jianying Zhang, Evandro F. Fang

**Affiliations:** 1 Department of Clinical Molecular Biology, Akershus University Hospital, University of Oslo, 1478 Lørenskog, Norway; helingwang.med@gmail.com (H.W.); s.h.lautrup@medisin.uio.no (S.L.); domenica.caponio@medisin.uio.no (D.C.); zhjianying@csu.edu.cn (J.Z.); 2 Xiangya School of Stomatology, Central South University, Changsha 410083, China; 3 The Norwegian Centre on Healthy Ageing (NO-Age), 0010 Oslo, Norway

**Keywords:** DNA damage, DNA repair, Alzheimer’s disease (AD), age-related disease

## Abstract

DNA repair ensures genomic stability to achieve healthy ageing, including cognitive maintenance. Mutations on genes encoding key DNA repair proteins can lead to diseases with accelerated ageing phenotypes. Some of these diseases are xeroderma pigmentosum group A (XPA, caused by mutation of *XPA*), Cockayne syndrome group A and group B (CSA, CSB, and are caused by mutations of *CSA* and *CSB*, respectively), ataxia-telangiectasia (A-T, caused by mutation of *ATM*), and Werner syndrome (WS, with most cases caused by mutations in *WRN*). Except for WS, a common trait of the aforementioned progerias is neurodegeneration. Evidence from studies using animal models and patient tissues suggests that the associated DNA repair deficiencies lead to depletion of cellular nicotinamide adenine dinucleotide (NAD^+^), resulting in impaired mitophagy, accumulation of damaged mitochondria, metabolic derailment, energy deprivation, and finally leading to neuronal dysfunction and loss. Intriguingly, these features are also observed in Alzheimer’s disease (AD), the most common type of dementia affecting more than 50 million individuals worldwide. Further studies on the mechanisms of the DNA repair deficient premature ageing diseases will help to unveil the mystery of ageing and may provide novel therapeutic strategies for AD.

## 1. An Overview of DNA Damage and DNA Damage Response (DDR)

### 1.1. DNA Damage and DDR

DNA carries genetic instructions for the development, functioning, growth, and reproduction of cells. DNA is inherently unstable due to both spontaneous chemical instability and modifications caused by either exogenous or endogenous agents causing DNA damage [1,2]. It has been estimated that each individual cell is subjected to up to one million DNA changes per day [3,4,5,6]. DNA damage is well known to affect both DNA replication, transcription, and a broad spectrum of signaling pathways including the nucleus to the mitochondria signaling pathway [1,7]. In this review, we update the progress of mechanistic studies on the key DNA repair pathways, including base excision repair (BER), nucleotide excision repair (NER), and double-strand break repair (DSBR). Furthermore, we focus on the role of DNA damage in ageing-related neurodegenerative diseases, with particular attention to its role in both rare premature ageing diseases, and the age-predisposed condition Alzheimer’s disease (AD).

Unlike other macromolecules of the cell, DNA cannot be replaced, but must be repaired to remain intact and functional. To avert deleterious consequences of DNA damage, cells have evolved several mechanisms, collectively termed the DNA damage response (DDR), to detect DNA damage, signal its presence and promote its repair. A variety of DDR pathways have been identified in organisms ranging from bacteria to humans, and are essential to life [8]. Three main repair pathways in mammalian neurons, including base excision repair (BER), nucleotide excision repair (NER), and double strand break repair (DSBR), are discussed below.

### 1.2. Major DNA Repair Pathways and Their Roles in Neurons and Microglia

**Figure 1:**
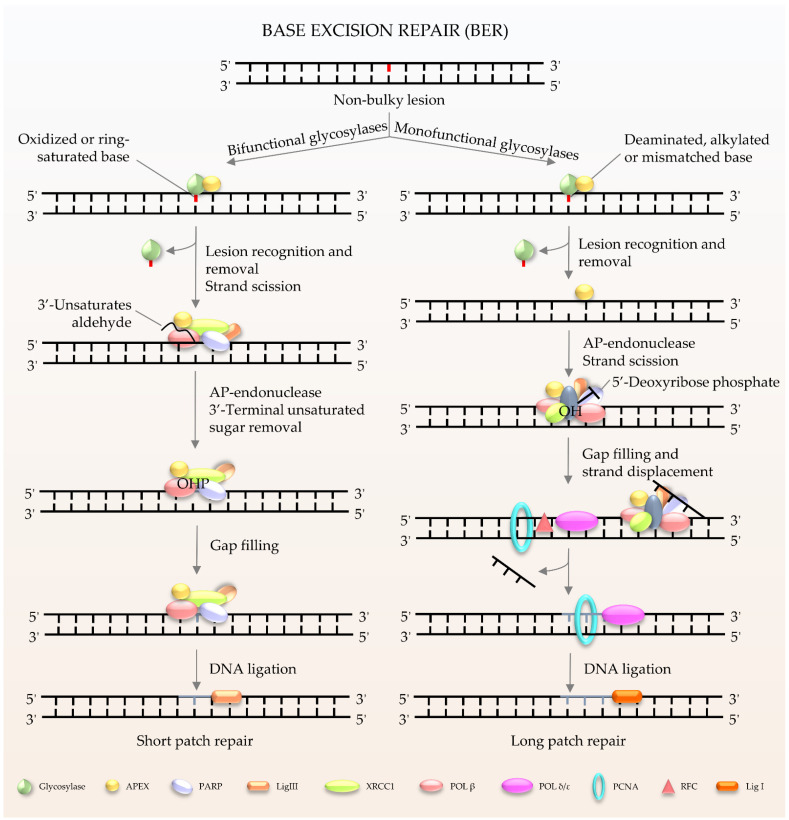
Schematic of BER in mammalian cells. BER is the key pathway to remove and repair damaged bases. It starts with a DNA glycosylase to recognize and eliminate the damaged base, creating an abasic site. The gap is finally filled by DNA polymerases. The short patch is repaired with a single nucleotide, and the long patch is synthesized with 2–13 nucleotides.

BER (Figure 1) is the major pathway involved in the repair of oxidative lesions and is responsible for the repair of non-bulky DNA oxidation, deamination, and alkylation [9,10,11]. The first step in the BER pathway uses a lesion-specific DNA glycosylase to recognize and eliminate damaged base pairs, which initiates the pathway [12]. Either a bifunctional or monofunctional DNA glycosylase catalyzes the cleavage of the *N*-glycosidic bond by flipping the damaged base out of the double helix to release a free base and create an abasic site (AP site). The repair is further processed by AP endonuclease 1 (APE1) to cleave the DNA backbone 5′ to the AP site, hereby producing 3′-hydroxyl and 5′-2-deoxyribose-5′-phosphate (5′-dRP). The gap is then filled by DNA polymerases using 3′-hydroxyl through template-directed synthesis via either short-patch or long-patch repair, depending on the number of inserted nucleotides. In the case of short-patch BER, DNA polymerase β (Polβ) and the inherent dRP-lyase activity of Polβ detaches the 5′-dRP to replace a single nucleotide. In general, long-patch repair requires the assistance of Polδ/ε and flap endonuclease 1 (FEN1) to substitute the displaced 5′-end structures with 2–13 nucleotides [10]. The importance of BER has been exemplified by the lethality of *Ape1^−/−^* or *Polβ^−/−^* mouse models. Deletion of mouse APE1 (also known as Apex1) leads to embryonic lethality, and deficiency in cells can promote cellular senescence and premature ageing features [13]. It has been demonstrated that Polβ defects can hamper amyloid β (Aβ)-induced neurogenesis in mice [14]. In particular, knockdown of Polβ inhibited the 42 amino acids form of Aβ peptide (Aβ_1–42_)-promoted differentiation of nestin^+^ progenitor cells into nestin^+^/Distal-less homeobox 2 (Dlx-2^+^) neuroblasts [14]. Moreover, pharmacological blockage of Polβ prevented Aβ_1–42_-induced differentiation of progenitors into MAP-2^+^ neurons [14]. It is worth noting that many proteins involved in BER not only exist in the nucleus, but also in the mitochondria [8]. Mitochondrial DNA (mtDNA) is more susceptible to the adverse effects of reactive oxygen species (ROS) produced during oxidative phosphorylation than nuclear DNA [15]. Thus, BER is considered to be the main repair pathway for DNA damage in mitochondria [16,17].

**Figure 2:**
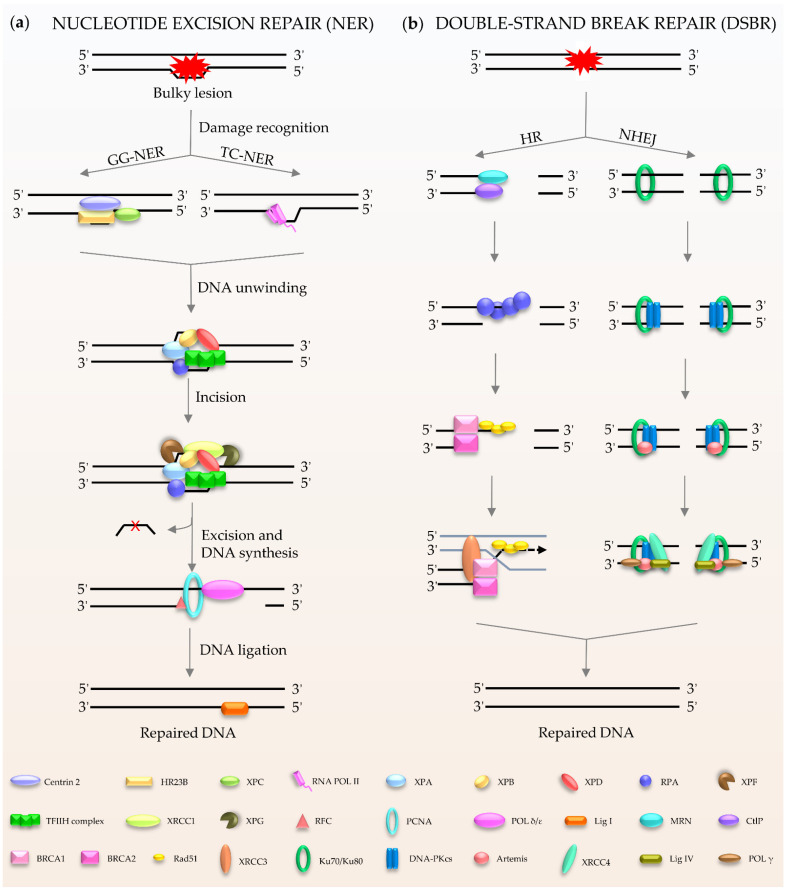
Schematic of NER and DSBR in mammalian cells. (**a**) NER is composed of GG-NER and TC-NER. XPC scans the double helix to identify the lesion, and forms a complex with centrins, HR23B among others, to induce NER activity. Subsequently XPA and TFIIH act as translocase and helicase to unwind the DNA. The DNA lesion is excised by XPF and XPG. Finally, the gap is filled by DNA polymerases and DNA ligase I. (**b**) DSBR consists HR and NHEJ. HR is initiated by MRN. Rad51 participates in the search for homologous copies, and its homologues are involved in DNA strand invasion and subsequent homologous recombination to repair. NHEJ is activated by Ku80/Ku70. The damaged ends are trimmed by artemis; after that, the gap is filled by the action of DNA polymerase. The final repair is mediated by DNA ligase IV and cofactor XRCC4.

NER (Figure 2a) is a central DNA repair pathway and a highly dynamic process responsible for removing bulky lesions from the genome [18,19,20]. In humans, NER is composed of two branches, global genome NER (GG-NER) [21] and transcription-coupled NER (TC-NER) [22,23], which are distinguished by their different recognition methods. XPC, as the main damage recognition factor, scans the double helix to identify the lesion, and forms a complex with centrins, HR23B, etc., to induce GC-NER activity [24]. In the case of TC-NER, it is primed by transcribing RNA polymerase II [25]. After recognition, the two factors share the same pathway for repairing the damage. The repair factor XPA and the large multi-subunit transcription factor IIH (TFIIH) act as translocase and helicase, respectively, to unwind the DNA [19,26]. The DNA lesion is then excised through double DNA nicks with two endonucleases, XPF and XPG. Finally, the gap is filled by Polδ/ε and ligase I [21,22].

Double-stranded breaks (DSBs) are the most harmful type of DNA damage in terms of genomic integrity [27]. Mammalian cells use two main DSBR pathways: homologous recombination (HR) and non-homologous DNA end joining (NHEJ) (Figure 2b) [28,29,30,31,32]. HR is the main method for DSBR utilized during embryogenesis and embryonic development [33]. The damaged ends of DNA are recognized by the Mre11-Rad50-Nbs1 (MRN) complex [34]. This complex performs extensive DNA processing, which together with CtIP generates 3′ single-stranded DNA (ssDNA). Rad51 participates in the search for homologous copies, and its homologues are involved in DNA strand invasion and subsequent HR to achieve highly accurate damage repair [35]. The cyclic heterodimer Ku70/Ku80 triggers NHEJ and then recruits DNA-PKcs [36,37,38,39]. Afterwards, nucleases (such as Artemis) deal with the damaged ends, and the gap is filled by DNA polymerases, such as Pol μ/λ/γ [40,41]. The final step of the repair pathway is mediated by DNA ligase IV and X-ray repair cross-complementing protein (XRCC4) [39,41]. It is well known that the process of DDR requires ATP, especially for DNA ligation. In humans, in response to DNA damage, cells mobilize more than ten thousand ATP molecules to repair just one DSB [42]. Interestingly, DNA ligase IV, the key enzyme of DSBR, uses NAD^+^ as a substrate for double-strand connection to mediate the final repair [43].

**Figure 3:**
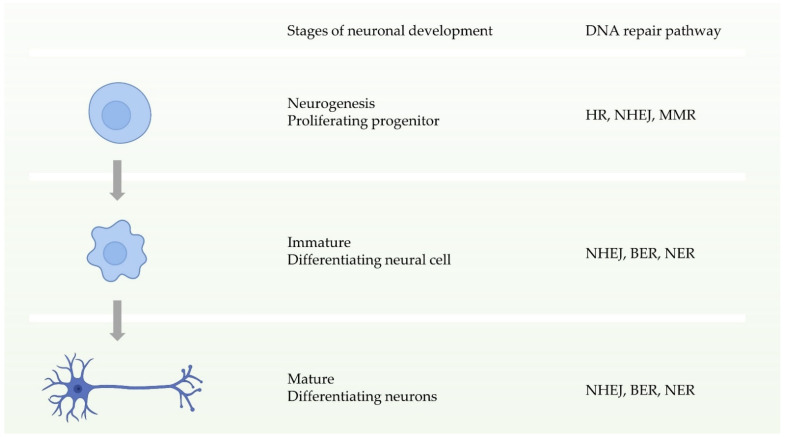
Schematic of the availability of different DNA repair pathways during different stages of neuronal development.

Neuronal homeostasis is a prerequisite for the development and function of the nervous system, and requires high fidelity and stable inheritance [44]. In normal cellular activities or DNA replication, the high precision and integrity of the genome must be maintained after DNA damage. Multiple DDR pathways in cells drive the biological functions ensuring this procedure. During the early stages of neuronal development, that is, neural progenitor proliferation, the nervous system has entire DDR pathways. In other words, it can repair double-strand breaks through two pathways of HR and NHEJ before neuronal maturation (Figure 3) [11,44]. In contrast, during neuronal maturation, NHEJ becomes the only way to repair double-strand break damage [45]. Defects in these DDRs can cause neurological disorders. Studies have shown that defects in NHEJ, NER, or BER increase risks to neurodegenerative disorders or neurodevelopmental defects [46,47,48,49,50,51].

Microglia are glial cells widely distributed in the brain and spinal cord. They are the main form of active immune defense in the central nervous system, and are key cells involved in the neuroprotective functions that maintain normal brain function; however, microglia can be hostile to neurons in disease conditions [52,53]. Studies have shown that DDR-related proteins have an impact on the activity, function, and survival status of microglia. In DDR, especially in DSBR, the deficiency of one crucial protein, ataxia-telangiectasia mutated (ATM) [54], results in abnormally active microglia, and stimulates excessive production of pro-inflammatory factors, which result in neurotoxicity [55]. More specifically, ATM dysfunction causes damage to DNA repair, and leads to the further accumulation of impaired cytoplasmic DNA. In microglia, cytoplasmic DNA can subsequently activate an antiviral defense system via the DNA sensor stimulator of interferon genes (STING). Cytoplasmic DNA can also trigger absent in melanoma 2 (AIM2) inflammasomes and, in parallel, induce elevated levels of cytokine precursors, such as pro-IL-1, through proteolytic processing. These processes create an extreme environment of neurotoxic inflammation [55]. In addition, the DNA excision repair protein, ERCC1, is very important for NER and DSBR [56]. Loss of ERCC1 results in microglial death and a compensatory increase in proliferation [57]. Of note, it is speculated that, in a mouse model of *Cx3cr1-Ercc1^ko/loxP^* and *Cx3cr1-Ercc1^wt/loxP^*, *Ercc1*-deficient microglia might have a link with ageing-related phenotypes [57].

## 2. Crosstalk between Nucleus and Mitochondria in DNA Damage

DNA damage not only accumulates in chromosomal DNA, but also in mtDNA, leading to mitochondrial dysfunction [58]. Dysfunctional mitochondria are targeted for lysosomal destruction through mitophagy and are recycled for cell utilization, as well as being degraded by the ubiquitin-proteasome system (UPS) [59]. Mitochondrial dysfunction and mitophagy defects are likely key features of age-related neurodegenerative disorders [60]. The accumulation of mtDNA damage and the reduction of mitophagy are also hallmarks of premature ageing diseases, such as XP and A-T [61,62,63]. Although organisms have a large number of responses to DNA damage, not only will DNA lesions increase during ageing, but the efficiency of DDR will also decrease [7,64,65,66,67]. Many accelerated ageing diseases, such as XP, A-T, CS, and WS, and neurodegenerative diseases such as AD, are closely related to the mutation of DDR proteins and DNA damage in both the nucleus and mitochondria [68,69,70,71,72].

**Figure 4:**
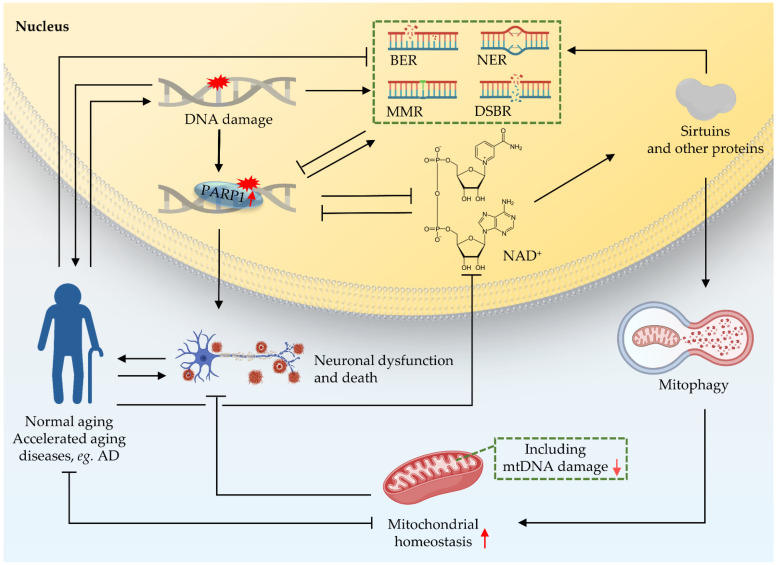
Crosstalk between nucleus and mitochondria in premature ageing and AD. Nuclear DNA damage can lead to mitochondrial dysfunction. One of the pathways through which the nucleus regulates mitochondrial function is the PARP1–NAD^+^–SIRT1 signaling pathway. NAD^+^ plays an important role as a reaction substrate in various pathways of DDR. Together, these contribute to ageing and the neurodegeneration in accelerated ageing. Black arrows indicate promotion, and inverted T bars indicate repression. Red arrows indicate up- or downregulation.

Crosstalk between the nucleus and mitochondria is essential for cellular function [7,63]; this crosstalk is a response to different ‘stimulators’ such as oxidative stress, DNA damage, and mitochondrial dysfunction [73,74]. There are accurate and rigorous regulatory mechanisms between the two organelles to control the stability of mitochondria [74]. One of them is that the nucleus regulates mitochondrial function through the poly-ADP-ribose polymerase 1 (PARP1)–NAD^+^–sirtuin 1 (SIRT1) signaling pathway (Figure 4). NAD^+^ is an important substrate for enzymes like PARPs and the NAD^+^-dependent deacetylases (sirtuins or SIRTs). NAD^+^ plays a key role in DDR. PARP1 monitors DNA lesions and subsequently recruits DNA repair proteins through PARylation, while consuming NAD^+^ [7,75]. PARP1 is continuously activated due to the accumulation of nuclear DNA damage. Hyperactivity of PARP1, as shown in multiple DNA repair deficient models (CS, XP), can lead to NAD^+^ depletion, thereby reducing the activity of sirtuins, and finally leading to mitochondrial dysfunction via impaired mitochondrial biogenesis and depleted mitophagy [76]. The sirtuin family shuttles between the nucleus, mitochondria, and cytoplasm in response to cell stimulation [74,77,78,79,80]. In addition, studies have shown that mutation of the *C. elegans pme-1*, the homologue of mammalian *PARP1*, increased NAD^+^ levels and Sir2.1 (the homologue of SIRT1 in *C. elegans*) activity, as well as increasing both healthspan and lifespan; mechanistically, this effect is at least partially contributed to by increased mitochondrial homeostasis through UPR^mt^ activation [63,68,81]. PARP1 interacts with SIRT1 to achieve signal transduction from the nucleus to the mitochondria [7]. Further studies on the role of PARP1–NAD^+^–SIRT1 signaling in nucleus-mitochondria crosstalk are necessary.

## 3. Impairment of the NAD^+^-Mitophagy Axis Is a Shared Mechanism in DNA Damage-Induced Accelerated Ageing Diseases and in AD

### 3.1. XPA

Xeroderma pigmentosum (XP) is a rare genetic disorder and is characterized by sensitivity to UV exposure [82,83]. Demographic data showed that the average age of skin cancer development in children with XP who do not use proper sun protection is less than 10 years old [83]. One in four of XP patients have accelerated neurological degeneration along with progressive neuron loss [83]. This disease not only shows skin symptoms, such as photosensitivity, pigment changes, etc., but also shows intractable neurological symptoms [84] such as sensorineural deafness, mental deterioration, and ataxia [85,86,87].

XP group A (XPA) (OMIM# 278700), a classic form of XP, is caused by mutations in the *XPA* gene (encoding the DNA damage binding protein XPA). XPA is also the first disorder shown to be caused by DNA repair defects [88] and is closely related to NER defects. The XPA protein plays a pivotal role in the NER pathway in which XPA can stimulate transcriptional factor TFIIH, as described earlier [89]. Together with the ssDNA binding protein RPA [20], TFIIH can coordinate the localization of endonucleases ERCC1-XPF [90] and XPG. XPA can also serve as a scaffold for repair proteins [91]. The interaction of XPA and ERCC1 can recruit the ERCC1-XRF complex and other complexes to the damaged sites on DNA [56,92,93,94].

Recent evidence suggests that XPA shows a significant mitochondrial phenotype in silico and in vivo [63]. XPA deficient cells show defective mitophagy with excessive cleavage of PINK1 and increased mitochondrial membrane potential. The mitochondrial abnormalities appear to be caused by decreased activation of the NAD^+^–SIRT1–PGC-1α axis triggered by hyperactivation of PARP1. The NAD^+^-dependent enzyme SIRT1 regulates PGC-1α, which in turn regulates uncoupling protein UCP2 [95,96]. The expression of UCP2 can regulate mitochondrial membrane potential and rescue XPA deficient cells from mitophagy defects [63]. Another important role of PGC-1α is that loss of this central transcription factor leads to neurodegeneration [97,98]. Additionally, PARP1 activation is well known as a major cause of neuronal death, and NAD^+^ depletion plays a role in PARP1 hyperactivation-mediate neuronal death [99]. The activation of PARP can also drive an accelerated ageing phenotype in XPA, while PARP inhibitors or compounds that increase cellular NAD^+^ can partially neutralize several ageing phenotypes [63]. The lifespan curtailed in the *xpa-1* worms can also be rescued by treatments with NAD^+^ precursors, such as nicotinamide riboside (NR), nicotinamide mononucleotide (NMN), or PARP inhibitor Olaparib [63]. XPA proteins are localized in the nucleus, but are not present in mitochondria, indicating that the underlying mechanism of defective mitophagy in XPA revolves around signaling from nucleus to mitochondria, and that mitochondrial defects may be a secondary response to nuclear DNA repair defects [100,101].

### 3.2. CS

Cockayne syndrome (CS) is a rare autosomal, recessive inherited segmental premature ageing syndrome caused by loss of function of either CS group B protein (CSB) or CSA. CS patients have an average life expectancy of 12 years and show, amongst other symptoms, cachectic dwarfism and severe neurological impairments [102,103]. The CS proteins are involved in transcription and DNA repair, including transcription-coupled NER and BER [104].

Up to 80% of CS patients suffer from prominent sensorineural hearing loss by the age of 10, one of the most prominent age-associated conditions [105,106]. Mouse models of CS (both *Csa^−/−^* and *Csb^m/m^*) reproduce this progressive hearing loss. NAD^+^ was reduced in the cochlea of CS mice, and 10 days treatment with NR rescued the progressive high-frequency hearing loss, improved outer hair cell survival, and normalized hearing capability [107]. NR treatment normalized the synaptic ribbons in the inner hair cells, which facilitated higher vesicle turnover, suggesting that the mechanism is similar to that underlying human hearing loss [107]. Interestingly, several studies have shown mitochondrial dysfunction and impaired mitophagy as a common feature of CS [63,108,109,110,111,112]. Restoration of mitochondrial function, via NAD^+^ augmentation or high-fat diet extended lifespan and improved the healthspan of CS mouse and *C. elegans* models, validating an important role for mitochondrial dysfunction in CS [63,110,112]. Additionally, microarray analyses of cerebellar samples from CS patients have confirmed signatures of dysfunctional mitochondria and compromised mitophagy/autophagy. Impaired mitochondrial homeostasis has also been shown in CS cellular and *C. elegans* models, which was also reversed by NAD^+^ augmentation [112].

The underlying mechanism of NAD^+^ depletion in CS likely centers around the nuclear-to-mitochondria crosstalk [7]. Increased DNA damage, caused by lack of CSA or CSB, activates the DDR regulated by PARP1. This PARP1-mediated decline of available NAD^+^ has been shown to decrease the activity of SIRT1 in CS [110], again leading to decreased mitophagy, likely via the depression of PGC-1α and UCP2, as is also seen in XP (described above) [63]. Additionally, microarray analysis of brain samples from CS patients shows the altered expression of proteins involved in mitophagy (ULK1, AMPK), possibly due to extensive DNA damage in the nuclear and mitochondrial DNA causing a decline of NAD^+^ and disruption of mitochondrial homeostasis [112]. Dysfunctional mitochondrial morphology and compromised mitophagy in both human cells and animal models confirm these findings [63,107,110,112].

### 3.3. A-T

Ataxia-telangiectasia (A-T; OMIM# 208900) is a neurological disorder of ataxia caused by biallelic mutation of *ATM* gene (OMIM# 607585). It is characterized by cerebellar Purkinje neuron degeneration, carcinogenesis, and immune dysfunction [113]. A-T patients exhibit childhood-onset cerebellar ataxia, abnormal extrapyramidal motility, immunodeficiency with repeated infections, a high risk of malignant tumors, etc. [114,115,116]. Patients with A-T usually lose motor function around the age of 10, and die at around the age of 20 or 30 due to malignant tumors or respiratory failure [116,117].

The *ATM* gene encodes ATM kinase (ATM), which is a PI3K family kinase. ATM is a DNA break-triggered kinase that plays a role in cell cycle regulation and DNA repair. The prominent role of ATM is in DDR [54], especially in the DSBR mechanism and cell redox balance. In A-T, the activation of microglia and neurotoxic cytokine secretion are caused by the accumulation of cytoplasmic single stranded/double stranded self-DNA which is recognized by the innate immune system due to DNA repair defects [118,119]. Recent studies give insight into this issue. Song et al. utilized *ATM*-deficient human fibroblasts and mouse models, verifying that microglia triggered cell-autonomous activation via the presence of ss/dsDNA in the cytoplasm in vitro and in vivo [55]. STING is a downstream adaptor protein of cGMP-AMP synthase (cGAS) in the cytosolic DNA-sensing pathway in innate immunity. The activation of STING leads to up-regulation of interleukin 1 (IL-1β) dependent nuclear factor κB (NF-κB) transcription and production of pro-IL-1β [55,120]. Song and co-workers further proved that pro-IL-1β is processed by caspase 1 in active inflammasomes, followed by the release of mature IL-1, causing synaptic degeneration and apoptosis [55].

In *Atm^−/−^* mice and *atm-1* worms, intervention with the NAD^+^ precursor NR, PARP1 inhibitor Olaparib, or SIRT1 activator SRT1720, alleviated A-T-related phenotypes with an obvious extension of lifespan and improved healthspan [68]. It is speculated that the underlying molecular mechanisms are (1) the three interventions mentioned above improved ATM-deficient phenotypes, at least in part via the NAD^+^/Sirtuins signaling pathway, (2) NAD^+^ replenishment improved mitochondrial quality via DCT-1-associated mitophagy, and (3) NAD^+^ supplement stimulated DNA repair through activation of Ku70 and DNA-PKcs [68].

### 3.4. WS

Werner syndrome (WS; OMIM# 277700) is a rare autosomal recessive, segmental progeroid syndrome caused by homozygous or compound heterozygous loss of function mutations in the *WRN* gene [121]. WRN protein belongs to the RecQ helicase family of proteins, which play critical roles in genome maintenance and which are often referred to as guardians of the genome [122,123,124]. WRN is unique among RecQ helicases in possessing both helicase and exonuclease activity [125]. Interestingly, unlike mammals, WRN activities are located on different proteins in *C. elegans* and *Drosophila melanogaster* WS models, leading to the conclusion that both exonuclease and helicase are essential for the etiology of the disease [70,126].

WS patients usually develop normally until they reach adolescence and therefore pathological characteristics are not apparent until the third decade of life [127]. The first sign is a lack of a growth spurt and a relatively short stature as adults. In the early third decade of life, patients begin to develop an aged appearance that includes skin atrophy, loss of subcutaneous fat, and graying and loss of hair. This is accompanied by a series of common age-related diseases that appear during middle age, including type 2 diabetes mellitus, hypogonadism, osteoporosis, atherosclerosis, and malignancies [128].

Due to this series of metabolic features, we speculated as to whether WS could be linked to mitochondrial dysfunction or NAD^+^ depletion. WS has been linked with many hallmarks of ageing, including mitochondrial dysfunction [70]. Additionally, 70% of WS patients develop diabetes, which could be caused by mitochondrial dysfunction [129]. We recently reported that NAD^+^ depletion is a major driver of the severe metabolic dysfunction in WS through dysregulation of mitochondrial homeostasis in WS human cells and *C. elegans* [126]. Moreover, WRN loss induced NAD^+^ depletion by affecting NMNAT1 levels, a key NAD^+^ biosynthetic enzyme, and 24 h treatment with NR corrected these defects. NAD^+^ precursor replenishment extended lifespan and increased healthspan, including the number of stem cells in both *C. elegans* and *Drosophila melanogaster* WS models [126].

Microarray analysis demonstrated that NAD^+^ precursor supplementation changes the transcriptomic profile related to metabolism, cellular stress, autophagy, and ageing in *C. elegans* and mice [68,130]. WRN dysfunction affects those pathways, especially mitochondria-related pathways such as fat metabolism and autophagy, while NAD^+^ repletion normalizes many of them at the transcriptional level, supporting its role in healthy longevity. Many studies support the correlation between compromised mitophagy and DNA repair deficient premature ageing disease [63,131,132]. In both WS human cells and *C. elegans*, NR supplementation increases NIX and ULK-1 dependent mitophagy [126].

In addition, *WRN* mutation leads to cellular NAD^+^ reduction through the upregulation of cellular NAD^+^ consumption, such as DNA-damaged induced PARP activation. In turn, it affects SIRT1 activity, leading to mitochondrial dysfunction and metabolism alternations [68,133]. NR administration increases HR in *C. elegans* by dramatically decreasing the numbers of RAD-51 cells in the mitotic region [126].

### 3.5. AD

AD is a progressive neurological disorder that is the most common cause of dementia, which affects more than 50 million people worldwide, a number expected to increase to more than 152 million by 2050 [134]. AD is characterized by behavioral abnormalities, progressive cognitive impairments, and memory loss. The pathological hallmarks of AD include Aβ plaques, neurofibrillary tangles (NFTs) consisting of hyperphosphorylated Tau protein aggregates, severe neuroinflammation, neuronal loss, and synaptic dysfunction [135,136]. Additionally, several studies have shown a central role for genomic instability, extensive DNA damage, and insufficient DNA repair in the disease [137]. It has been demonstrated that decreased function of the DNA repair pathway resolving oxidative DNA damage, the BER pathway, plays an important role in AD pathogenesis [138,139,140,141]. Both protein level and activity of key BER proteins including Polβ, uracil glycosylase, and 8-oxoguanine DNA glycosylase 1 (OGG1), are downregulated in AD brains [142,143]. Polβ especially seems essential in AD progression; reduced Polβ protein expression and activity has been demonstrated in early disease stages, and further declines with disease progression [139,144]; the Bohr laboratory showed that lack of Polβ exacerbates AD progression in a Polβ deficient AD mouse model [144,145]. Complete loss of Polβ in mice also leads to embryonic lethality, and gross brain developmental impairments including lack of the olfactory bulb. Polβ knock out cells are also hypersensitive to DNA damage and exhibit mitochondrial dysfunction [71,72].

Oxidative DNA damage is prominent in the mitochondria, and both excessive ROS and mitochondrial dysfunction have been demonstrated in AD, supporting the importance of DNA damage and repair. Furthermore, together with co-workers, we have demonstrated for the first time that compromised mitophagy is a central feature likely affecting both initiation and progression of AD (reviewed in [146,147]).

NAD^+^ levels are lower in postmortem human and AD mouse brains compared to controls [145]. Treatment with NAD^+^ precursors of AD mouse models can alleviate several features of AD including Aβ and Tau pathologies, neuroinflammation, cognitive impairments, and synaptic dysfunction, at least partially, through activation of mitophagy, thereby preventing the cells from accumulating damaged mitochondria [145,148]. We and co-workers showed that NR treatment of Polβ deficient AD mice normalized the level of DNA damage to that of WT mice. Additionally, NR treatment increased NAD^+^ in the brain which restored mitochondrial dysfunction and oxidative stress via a pathway likely involving decreased PARP1 activity, increased sirtuin activity, and induced mitophagy [145]. Boosting NAD^+^ levels resulted in improvement of AD characteristics including neuronal plasticity and function, cognitive impairments, and neuroinflammation [145]. In conclusion, several studies have linked DNA repair, NAD^+^, and mitophagy, but the exact mechanism explaining how NAD^+^ regulates DNA repair pathways and how mitophagy is activated remain elusive and targets for future studies.

## 4. Conclusions and Future Perspectives

DNA repair plays a fundamental role in life and health, while dysfunctional DNA repair drives or increases risks of premature ageing diseases, and a broad spectrum of other diseases, such as AD. In this review, we have updated recent progress in description of the major DNA repair pathways, including BER, NER, and DSBR. Mutations of genes involved in these pathways cause a group of premature ageing diseases, such as XPA, CS, A-T, and WS. Recent studies suggest DNA damage increases the risks of AD. Emerging questions and perspectives include (a) how cells orchestrate different DNA repair pathways to maintain genomic stability and health; (b) why is there little to no neurodegeneration in WS, while DSB is detrimental to neurons; and (c) research to find clinical evidence supporting promising drug candidates should be undertaken. Further studies on the mechanisms of DNA repair and their roles in healthy ageing and brain maintenance will shed light on the development of novel interventional strategies and treatments to support a healthier lifespan.

## Data Availability

Not applicable.
